# Characterization of the Global DNA Methylome in *Escherichia coli* Under Silver Stress

**DOI:** 10.3390/microorganisms14102369

**Published:** 2026-10-16

**Authors:** Anuoluwapo Anele, Sunday O. Peters, Malarmathi Muthusamy, Joseph L. Graves, Misty D. Thomas

**Affiliations:** 1 Department of Biology, North Carolina Agricultural and Technical State University, Greensboro, NC 27411, USA; gravesjl@ncat.edu; 2 Department of Animal Science, Berry College, Mount Berry, GA 30149, USA; speters@berry.edu; 3 Department of Animal Genetics and Breeding, Veterinary College and Research Institute, Tamil Nadu Veterinary and Animal Sciences University (TANUVAS), Namakkal 637002, Tamil Nadu, India; malarmathi.m@tanuvas.ac.in

**Keywords:** DNA methylation, epigenetics, bacterial adaptation, two component response systems, cusS/R, silver, bacterial stress, adenine methylation, 6mA, 4mC

## Abstract

Bacteria are continually exposed to stressors, making their ability to sense, acclimate and adapt to changing conditions essential for fitness. Two-component response systems (TCRS) play a primary role in mediating these responses. However, the role of epigenetic modifications in acclimation and adaptation is not well understood. We characterized N6-methyladenine (6mA) and N4-methylcytosine (4mC) DNA methylation patterns in wild-type *Escherichia coli* K-12 MG1655, four *cusS* mutants and six silver-adapted mutants with and without exposure to 50 ng/mL AgNO_3_ for 1 h using Single-Molecule Real-Time sequencing. The *cusS* mutants were selected to study the impact of the mutations on DNA methylation. Global mean 6mA fractions were high across all strains with and without silver and were largely driven by the dominant GATC motif in all strains and the AACN_6_GTGC/GCACN_6_GTT motif in wild-type and silver-adapted mutants, while global mean putative 4mC fractions were lower. Across conditions, variation was observed in extended degenerate motifs with moderate-to-low methylation. Our findings suggest that motif-specific methylation patterns vary across silver exposure and genetic backgrounds, while global methylation levels in *E. coli* remain remarkably resilient. This study improves understanding of microbial stress adaptation and stress response, raising the possibility that methylation contributes to TCRS-mediated stress tolerance.

## 1. Introduction

Bacteria are constantly exposed to a variety of stressors, and their ability to sense, acclimate and adapt is critical to their fitness. These responses are mediated primarily through two-component response systems [1,2,3,4], which control processes such as antibiotic resistance, cell division and metabolism [1]. The typical two-component response system is composed of two proteins: a histidine kinase that detects external stimuli in the extracellular space, then relays this message via autophosphorylation and subsequent transphosphorylation to the second protein, the response regulator [1,2,3,4]. This response regulator then initiates transcriptional responses that position the cell to acclimate to the external stimuli [1,2,3,4]. In *Escherichia coli*, silver acclimation, adaptation and resistance have been most consistently associated with the CusS/R two-component system [4,5,6]. In this two-component system, CusS is activated after sensing Ag^+^, after which the phosphoryl group is transferred to CusR, which then activates a response transcription of *cusRS* and *cusCFBA* genes that leads to Ag^+^ efflux from the bacterial cell [4,5,6,7,8]. Silver is a potent, broad-spectrum antimicrobial that has been used for centuries [9,10]. Despite this characteristic, bacterial resistance to silver has been reported in the literature, and the experimental evolution of silver resistance has been shown to occur rapidly, making it an ideal antimicrobial environment to study bacterial stress, acclimation and adaptation responses [11,12].

Previous work has examined two-component response systems in bacteria across multiple contexts, including environmental effects on susceptibility and resistance [3,13,14]; genotype-by-environment interactions [4,15]; strain-dependent variation in system activity [4,8,16]; transcriptomic characterization of system-mediated responses [4,16,17]; epistasis within these systems [8]; and mutations in two-component response systems as drivers of acclimation and adaptation [4,8,18]. However, the role of DNA methylation in bacterial response to stress and the extent to which it contributes to strain-specific or acclimation-associated differences, particularly with respect to two-component response systems, remains unclear.

DNA methylation is an epigenetic modification involving the covalent addition of a methyl group to specific nucleotide bases, which can influence genome function without altering the underlying sequence [19,20,21]. It is present in all domains of life and contributes to diverse cellular processes, including gene expression, DNA repair, regulation, cell-cycle control, virulence and genome defense [19,22,23]. In bacteria, the three major forms of DNA methylation are N6-methyladenine (6mA), N4-methylcytosine (4mC) and 5-methylcytosine (5mC) [24,25,26]. Advances in long-read sequencing have enabled genome-wide characterization of bacterial methylomes, allowing identification of methylated sequence motifs and comparison of methylation patterns across strains, treatments and environmental conditions, providing foundational datasets for understanding epigenetic diversity, methyltransferase activity, strain-level variation and stress-associated responses.

In *Escherichia coli*, DNA adenine methyltransferase (Dam) accounts for the vast majority of 6mA, methylating adenine within 5′-GATC-3′ sites [19,27]. The Dam methylation system has been characterized genome-wide in *E. coli* K-12, including strain MG1655 [28,29]. In *E. coli*, Dam-mediated adenine methylation at GATC sites performs functions beyond DNA modification itself, where it contributes to mismatch repair, control of chromosome replication initiation and locus-specific transcriptional regulation [19,30,31]. Although Dam-mediated GATC methylation is well established in *Escherichia coli*, genome-wide methylome characterization remains important for placing this canonical system within the broader methylation landscape of related strains and environmental conditions. Bacterial methylomes may include both highly conserved core motifs and more variable or putative motifs associated with restriction-modification systems, strain background, adaptation or stress response. Comparative methylome profiling therefore provides an opportunity to determine whether dominant methylation systems remain stable across related genetic backgrounds while also identifying motif-specific variation that may not be apparent from Dam-focused analyses alone. A well-studied system can still serve as a biologically meaningful benchmark when comparing mutants, adapted lineages or stress-treated samples and gives a broader methylome resource than a Dam-only study.

Despite growing interest in bacterial epigenetics, the DNA methylation landscape associated with bacterial acclimation is poorly understood. In particular, dominant methylated motifs, strain-specific methylation patterns, stress-associated methylation shifts or comparative methylome data have not been systematically examined in wild-type *E. coli*, its single-nucleotide mutants and silver-adapted populations. In this study, we used mutants that had been generated via pORTMAGE recombineering harboring single-nucleotide mutations in *cusS,* the gene encoding the histidine kinase CusS in *Escherichia coli* K-12 MG1655 [5,12,32], alongside silver-adapted mutants evolved from the same ancestral strain in the presence of silver [7,8]. While two-component response systems are known to help bacteria translate environmental stimuli into stable or altered regulatory states, DNA methylation may represent an additional regulatory layer that reinforces, buffers or operates in concert with or independently of TCRS-driven transcriptional responses. Indeed, stress-associated methylation responses have been observed in bacteria with localized or motif-specific methylation plasticity occurring rather than genome-wide methylome disruption. Examples include localized methylation changes in *E. coli* under nutrient limitation [27], in *Salmonella enterica Typhimurium* under oxidative stress [31] and *Helicobacter pylori* under acid stress [33]. Specifically, Westphal et al. show that genome-wide GATC remains essentially fully methylated in *E. coli K-12 W3110* despite prolonged physiological stress under limited nutrient conditions [27]. However, approximately 0.18% of GATC sites (66 out of 37,452) showed detectable temporal dynamics [27]. These changes were not all in the same direction, with increases or decreases in change varying with the growth stage of the bacteria [27]. Zhang et al. also report motif-wide methylation changes in *Salmonella enterica Typhimurium* over a time course of oxidative stress while genome-wide methylation remained largely unchanged [31]. For example, through a time course of 10 min to 4 h under oxidative stress, the largest dip in GATC methylation was a 0.4% decrease from 99.8% to 99.4%, followed by recovery and almost complete methylation at 99.9% after 4 h [31]. Other 6mA motifs were either completely stable or showed similar transient dips before recovery [31]. Degenerate CCAGG-associated putative 4mC motifs also varied in presence or sequence under the same conditions [31]. Similarly, many 6mA motifs, including GATC in a *Helicobacter pylori* acid stress study were extraordinarily stable and were essentially acid-insensitive [33], whereas selective methylation was observed with HsdM1 Type I 6mA bipartite motifs [33]. For example, (HH)TCAVN_6_TGY was not detected at pH of 5 but was methylated at 41.2% at a pH of 7 [33]. And the 6mA motifs (H)TCAVN_6_TGY and AGTN_6_GACA were methylated at 35.7% and 44%, respectively at a pH of 5 but were not detected at a pH of 7 [33].

Therefore, the objective of this study was to characterize the genome-wide DNA methylation landscape of wild-type *E. coli*, four *cusS* mutants and six silver-adapted mutants using Single Molecule Real-Time (SMRT) sequencing, with an emphasis on identifying the dominant sequence motifs, shifts in motif distribution and comparative methylation patterns across our strains and genotypes under low-level silver stress. A low-level silver exposure was selected to impose a sub-lethal stress while maintaining bacterial growth, thereby enabling assessment of methylome responses under non-lethal exposure conditions. The experimental dose and exposure rationale are described in Section 2.2. SMRT sequencing is capable of detecting the three major bacterial DNA methylation types from native DNA, including 6mA, 4mC and 5mC, through polymerase-kinetic signatures [24,34]; hence, our choice of this platform for this study. However, its detection of 5mC is considered weaker since 5mC produces weaker kinetic signatures [26,35], while other authors note that this hurdle can be resolved using higher sequencing coverage (>250×) [36] or trained models [37,38]. Therefore, in this present study, we focused on SMRT-detectable 6mA and putative 4mC methylation and did not attempt comprehensive characterization of 5mC. This methylome characterization was designed to establish a baseline resource for future mechanistic investigation and to advance understanding of how DNA methylation contributes to bacterial acclimation and adaptation across distinct genetic backgrounds and stress environments.

## 2. Materials and Methods

### 2.1. Bacteria Strains

The wild-type *Escherichia coli* K-12 MG1655 (ATCC #10798D-5, Manassas, VA, USA), four *cusS* mutants (R15L, T14P, T17P and N279H) [32] and six silver-adapted mutants (SAM1, SAM2, SAM3, SAM4, SAM5 and SAM6) [7,8] were used in this study. The *cusS* mutants were previously constructed by introducing single-nucleotide substitutions into *cusS*, the gene encoding the histidine kinase CusS, via pORTMAGE recombineering in *E. coli* K-12 MG1655 [32]. The six silver-adapted mutants were generated through experimental evolution in silver nitrate as previously described [7,8]. The same *cusS* mutants used in this study were previously subjected to whole-genome sequencing in earlier work [32]. Whole-genome sequencing was also performed on the silver-adapted mutants (SAMs) [7,8] prior to this study. The e14 region was not specifically examined in these earlier analyses, so its status in the earlier stocks is not known. All strains from previous work were stored in 20% (*v*/*v*) glycerol at −80 °C.

### 2.2. Bacterial Culturing

All strains were revived from −80 °C glycerol stocks in Luria–Bertani (LB) broth (Invitrogen, Carlsbad, CA, USA) and grown overnight at 37 °C and 200 rpm in biological triplicate. The following day, each replicate was sub-cultured 1:10 into Davis Minimal Broth (DMB; ThermoFisher, St. Louis, MO, USA) supplemented with 0.1% (*w*/*v*) dextrose (Fisher Scientific Company, Fair Lawn, NJ, USA) as a carbon source and incubated at 37 °C and 200 rpm. On the third day, each replicate was sub-cultured 1:10 again into fresh DMB supplemented with 0.1% (*w*/*v*) dextrose and grown under the same conditions, this time in two parallel flasks, one designated for silver-free growth and one for silver-exposed growth. This three-day acclimation protocol and the dilution ratios were selected because direct inoculation into DMB from glycerol stocks resulted in incomplete or non-uniform revival across strains, while the stepwise approach of preconditioning yielded robust and consistent growth across all eleven strains.

On day three, the preconditioned cultures were grown to an OD_600_ of 0.2, corresponding to the mid-log phase in DMB for these strains, a condition also used in previous studies [8,39]. At this point, silver-free replicates were incubated for an additional hour. Silver-exposed replicates were supplemented with 50 ng/mL silver nitrate and incubated for one hour under the same conditions. Exposing the bacteria to silver nitrate at an OD of 0.2 over this period also allowed for a sufficient period of exposure while cultures remained in the log phase. We chose 50 ng/mL for our study because it was the original selection concentration for our strains [7,12] and had also been used in prior transcriptomic work [8] in our research group. We also chose one hour of exposure to silver because we did not want the bacterial cells to go through multiple rounds of cell division, which could lead to selection. The one-hour silver exposure time also matches the exposure time used in prior transcriptomic work [8] from our research group. All cultures were then pelleted by centrifugation at 4000× *g* for 10 min and stored at −80 °C. In total, 66 pellets were collected: three biological replicates per strain × 11 strains (wild-type, four *cusS* mutants, and six silver-adapted mutants) × two treatment conditions (silver-exposed and silver-free).

### 2.3. DNA Extraction, SMRT Sequencing and Modified Base Detection

All 66 frozen bacterial pellets were shipped on dry ice to SeqCenter (Pittsburgh, PA, USA, seqcenter.com) for DNA extraction, library preparation and primary bioinformatic processing. All samples were submitted as *Escherichia coli;* however, identifiers linking samples to specific strains, genotype classes (wild-type versus mutant versus adapted populations) and biological replicate groupings were withheld at the time of submission. Furthermore, experimental conditions were not provided. The service provider therefore conducted analyses blinded to sample-specific biological identity and experimental grouping. Briefly, genomic DNA was extracted using the PacBio NanoBind PanDNA kit (PacBio, cat. no. 103-260-000, Menlo Park, CA, USA). Sequencing libraries were prepared according to the “Preparing Whole Genome Libraries Using the HiFi Prep Kit 96” protocol using the SMRTbell^®^ Barcoded Adapter Plate 3.0 (PacBio, Menlo Park, CA, USA) and pooled libraries were bound and cleaned up using the Revio SPRQ Polymerase Kit (PacBio, Menlo Park, CA, USA). Sequencing was performed on the PacBio Revio platform (PacBio, Menlo Park, CA, USA) using HiFi chemistry. Demultiplexing, quality control and adapter trimming were performed using Lima 2.10.0. Genome assemblies were generated using Flye 2.9.2 [40] with the pacbio-hifi read input flag and a read error rate cutoff of 0.02. Assemblies were also circularized using Circlator 1.5.5 [41], annotated using Bakta 1.8.1 [42] and assembly quality assessed using QUAST 5.2.0 [43]. Methylation states of the assembled genomes were called from per-read kinetic data (interpulse duration ratios) embedded in PacBio HiFi reads. Strand-specific kinetic data were extracted using ccs-kinetics-bystrandify from the pbbam toolkit 2.1.0 and mapped to each assembly using pbmm2 1.14.0 (smrtlink-release_13.1.0.221970). Methylation calls were generated using the ipdSummary 3.0 algorithm (PacBio, Menlo Park, CA, USA) and putatively classified as 6mA or 4mC at positions meeting the default detection threshold (q-value ≤ 0.01). The PacBio ipdSummary model was run with default parameters. In the analysis used for our dataset, ipdSummary classified modification signals as putative 6mA or 4mC, which are the native DNA polymerase signatures it is designed to identify. Therefore, 5mC was not included as a callable modification class and 5mC calls were neither suppressed during analysis nor generated and discarded.

### 2.4. Comparative Methylome Analysis

The authors conducted statistical comparative methylome analyses to assess group differences, methylation distributions, and patterns across strains and experimental conditions, as described in Section 2.5. Methylation profiles were compared across samples by examining the presence or absence of motifs, motif-specific methylation fractions, and the number of methylated sites per motif.

### 2.5. Statistical Analyses and Data Visualization

All statistical analyses were performed in GraphPad Prism v11.0.1 (GraphPad Software, Boston, MA, USA), with significance defined as *p* < 0.05. Wild-type *E. coli* in the absence of silver served as the reference control for all comparisons. Because methylation fractions are bounded and not assumed to be normally distributed, non-parametric tests were applied throughout. Chi-square tests were used to assess whether the distribution of global methylated motif counts differed between each strain and the control. Mann–Whitney U tests were used for direct pairwise comparisons of global methylation fractions between each strain and the control. Kruskal–Wallis tests with Dunn’s post hoc multiple comparisons correction were used to assess overall differences in global methylation fractions across all strains simultaneously and to identify which specific pairs differed. Kruskal–Wallis testing was used as the primary omnibus nonparametric analysis across strain-condition groups, with Dunn’s multiple-comparisons procedure applied to compare each group with the wild-type without silver control, while controlling for family-wise type I error. Mann–Whitney U tests were performed separately as secondary pairwise comparisons between each of the strains, with and without silver and the same control. These Mann–Whitney *p* values were reported without multiple-comparisons adjustment because none reached nominal significance. Consequently, multiplicity adjustment would not alter their interpretation. The Mann–Whitney analyses were therefore used as descriptive pairwise comparisons rather than as the primary basis for inference across groups. For both fraction-based tests, global methylation fractions were summarized as weighted means across motifs within each biological replicate, where genome-wide weighted methylation fraction equals the sum of each motif’s methylation fraction multiplied by the number of genomic sites for that motif, divided by the total number of genomic motif sites across all motifs. This weighting approach was applied to avoid bias from unequal motif representation across samples. Count-based and fraction-based analyses were treated as distinct because they describe fundamentally different aspects of the methylome: motif counts reflect occurrence and enrichment, while fractions reflect the proportion of sites methylated. Motif presence or absence was analyzed based on methylated fractions, and motifs absent in a given strain were not encoded as zero in fraction-based analyses using Mann–Whitney U and Kruskal–Wallis tests. A motif was considered dominant/high in our dataset if the methylated fraction was >0.9, it had an IPD ratio of >4, was present across all three replicates of a strain, and was present across multiple strains. Moderate methylation/subdominant motifs were inferred when the methylated fraction was between 0.5 and 0.9, it had an IPD ratio of >1 and occurred across at least two replicates and/or strains. A motif was considered to have low methylation fractions and was considered a minor motif in our dataset if the methylated fraction was <0.5, it had an IPD ratio of >1, and was present in at least one replicate or strain.

Motif-specific methylation patterns were visualized as heat maps generated in GraphPad Prism v11.0.1. For each motif within each strain, methylation fractions across the three biological replicates were averaged to produce a single mean value for display. Cell color was scaled to the mean methylation fraction, with white indicating no detected methylation and progressively darker shading indicating higher methylation and reproducibility of each methylation across all replicates of a strain and treatment. Separate heat maps were generated for 6mA- and 4mC-associated motifs to facilitate comparison across strains and treatment conditions. Summary plots were also generated in GraphPad Prism. Shared and unique motifs were summarized across genotype groups and dominant motifs were identified for each condition.

## 3. Results

### 3.1. Sequencing Output, Methylome Data Quality and Overview of the Methylation Landscape

Sequencing and methylome quality were assessed using motif-level coverage, modification confidence scores, methylated-site fractions and reproducibility across biological replicates (Tables S1 and S2). Other kinetic quality metrics such as modification type, genomic instances of a modified motif, mean score, mean coverage and objective score are also included in Table S2. The genome size measured was approximately 4.64 Mb, which was an expected size for *E. coli*. The sequencing depth was also rich, at >400× mean coverage and % bp ≥ Q30 of at least 97% (Table S1). Detected motifs were interpreted as high-confidence only when supported by adequate coverage, consistent detection across replicates and SMRT confidence metrics. Only data with mean IPD ratio of >1 was used for analysis and data interpretation. At such thresholds, the methylation-calling software is detecting kinetic perturbations and useful information rather than just random flat noise. In addition to 6mA and putative 4mC methylation types, additional polymerase-kinetic signals were reported as modified bases. This means that the chemical identities of these bases could not be assigned using our standard native-DNA SMRT methylation workflow. These signals could not be classified as 6mA, 4mC, 5mC or any other specific modification type. Hence, since their modification chemistry could not be determined with confidence via SMRT sequencing and subsequent methylation calling, we did not make any further inferences, analyses or interpretations from such data. Furthermore, in our study, one replicate of SAM6 (with silver, replicate 1) assembled into two contigs. SAM4 (without silver, replicate 2) had a normal-length assembly compared to the other five SAM4 assemblies, which were about 15.2 kb shorter because they lack e14. These outliers have been addressed in detail in the discussion and Tables S1 and S2.

### 3.2. Comparative Methylation Patterns Across Strains or Conditions

Global genome-wide methylation profiling revealed that 6mA represented the predominant modification category detected across our dataset. Counts of globally methylated 6mA motifs in each strain were compared with the wild-type in the absence of silver (which served as control), using the chi-square test to assess motif enrichment patterns (Table 1). For our first chi-square analysis of global genome-wide 6mA motifs, N was the total pooled motif counts across all replicates and motifs per strain. This was intended as a distribution-level enrichment test per strain rather than tests of replicate-level biological variation. Chi-square tests showed that pooled motif counts of all strains differed significantly from the control (*p* < 0.05). Because chi-square is highly sensitive to sample size, we also included effect size measures in our analyses to show how meaningful the significant difference is. The effect size (Cramer’s V) was weak (0.13–0.14) for all *cusS* mutants and negligible (<0.1) for all SAMs. Therefore, although the chi-square test was statistically significant, the effect size indicates that the difference in motif distribution was detectable but weak in magnitude. Subsequently, we did another chi-square analysis of global genome-wide 6mA motifs for each replicate of each strain without pooling across replicates to test for replicate-level biological variation (Table S3). Here, N was the total pooled motif counts of each replicate of a strain. Again, the results show that motif counts of all strains differed significantly from the control (*p* < 0.05). However, the effect sizes were also weak (0.13–0.14) for all *cusS* mutants and negligible (<0.1) for all SAMs, showing that the detected difference in motif distribution was small. The consistently larger effect sizes in the *cusS* mutants reflect their greater motif compositional divergence from the wild-type control, particularly the complete absence of the AACN_6_GTGC/GCACN_6_GTT motif, which remained detectable in the SAMs.

For our first chi-square analysis of 4mC motifs, N was the total pooled motif counts across all replicates and motifs per strain. This was intended as a distribution-level enrichment test per strain rather than tests of replicate-level biological variation. Our results showed that global putative 4mC patterns showed selective significant shifts (*p* < 0.05) in methylated motifs when compared to the control (Table 2). These shifts were associated with mutation and stress, with these changes more evident in silver-adapted mutants than in *cusS* mutants. Specifically, the chi-square test showed significant differences between the control and SAM1 without silver stress, the control and SAM2 without silver stress and the control and SAM4 without silver stress. The chi-square test also showed significant differences between the control and R15L with silver stress, the control and SAM1 with silver stress, the control and SAM3 with silver stress, the control and SAM5 with silver stress and the control compared to the wild-type with silver stress. Also, to account for the sensitivity of chi-square to sample size, effect size measures were calculated for 4mC motifs. Statistical significance was interpreted alongside Cramer’s V or phi coefficient for effect size measurements. Because 4mC motifs in our dataset were not as diverse as the 6mA motifs, the effect sizes for chi-square analyses were reported as Cramer’s V for contingency tables larger than 2 × 2 and as the phi coefficient for 2 × 2 comparisons, consistent with standard reporting conventions for categorical association tests. These results showed that for associations between control and strains that were significant, the effect size (Cramer’s V or phi coefficient) was negligible (<0.1) for all strains, indicating that detected differences in motif distribution were weak in magnitude. To test for replicate-level biological variation, we also did the chi-square analysis of global genome-wide 4mC motifs of each replicate of each strain (Table S4). Here, N was the total pooled motif counts of each replicate of a strain without pooling across strains. Results showed select statistical significance for one replicate of SAM1 without silver treatment, one replicate of SAM2 without silver treatment, one replicate of SAM4 without silver treatment and one replicate of SAM3 with silver treatment. All effect sizes were negligible (<0.1) for all these SAMs, showing that the detected difference in motif distribution was very weak.

The Mann–Whitney U tests, however, revealed that global methylation levels in all strains were stable with and without silver and no significant differences were determined when compared to the control (*p* > 0.05) (Table S5). Global weighted mean 6mA methylation fractions ranged from ~0.69 to nearly 1.00, showing consistently high methylation levels throughout all our strains under our conditions (Table S5). Global weighted mean putative 4mC methylation fractions ranged from ~0.08 (Table S7) to nearly 0.09, indicating a reproducible low-level methylation pattern rather than stochastic or sample-specific variation.

**Figure 1:**
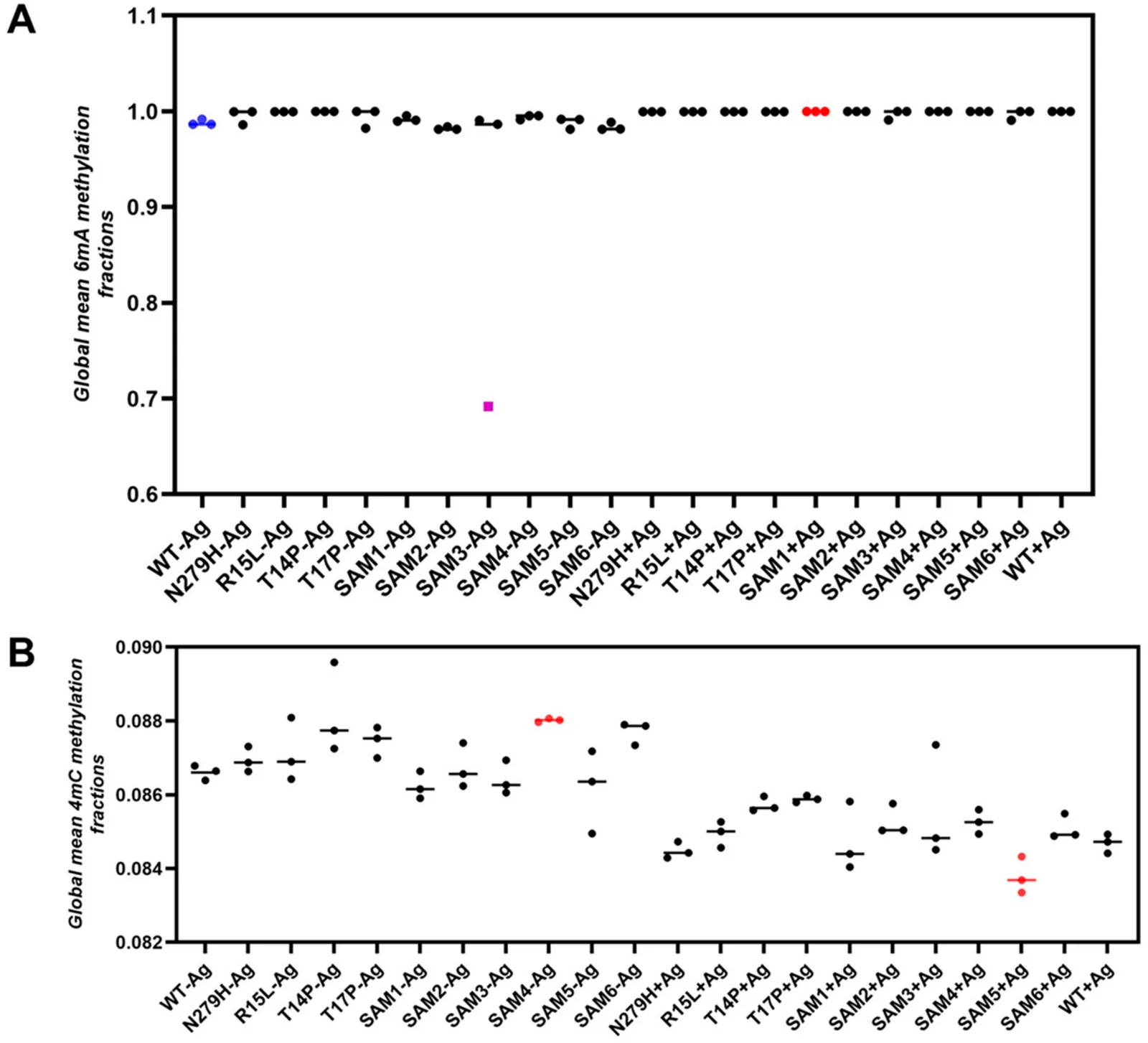
Comparison of global methylation fractions across strains and treatment conditions. Graph showing global weighted mean methylation fractions for (**A**) 6mA and (**B**) putative 4mC in wild-type and mutant strains without and under silver stress conditions. Individual points represent biological replicates for each strain and central lines indicate group medians. In (**A**), blue color-coded points indicate all three replicates of the control (wild-type without silver) and red color-coded points indicate all three replicates of SAM1 with silver, which were significantly different from the control. Also in (**A**), one replicate of SAM3 without silver exposure whose global methylation is much lower than other 65 samples is emphasized in a purple color-coded square point. In (**B**), replicates that differ significantly from each other are red color-coded. Statistical significance (*p* < 0.05) across groups was assessed using the Kruskal–Wallis test with Dunn’s post hoc multiple-comparisons analysis.

We also performed Kruskal–Wallis tests to assess global methylation fractions across all strains and replicates compared to the control (Figure 1). Generally, there was a slight upward shift in global methylation across silver-exposed strains compared to untreated strains. This partly reflects fewer low-fraction degenerate motifs being detected in treated samples, which raises the weighted mean. Also, interestingly, global methylation across replicates was more consistent after silver stress compared to strains not exposed to silver. The analyses also revealed that global 6mA methylation fractions showed a significant strain effect (*p* < 0.05) relative to the unstressed wild-type (Table S6). Post hoc comparisons using the Dunn test identified SAM1 under silver exposure as significantly different from the untreated wild-type control (adjusted *p* = 0.0344) (Table S6). However, the corresponding pairwise Mann–Whitney U comparison was not significant (*p* = 0.1, Table S5). These results should be interpreted within their respective statistical frameworks (explained below at the end of Section 3) with the significant Dunn’s comparison not necessarily indicating a substantial difference in methylation fraction. The negative mean-rank difference of −49.33 of SAM1 indicates that it had higher ranked methylation-fraction values than the control (Table S6). SAM1 also showed the most negative mean-rank difference among the comparisons, indicating the strongest upward shift in ranked methylation-fraction values relative to the control and suggesting that SAM1 contributed most strongly to the overall Kruskal–Wallis significance (Table S6). The Kruskal–Wallis test for global putative 4mC also revealed a significant overall difference among groups (*p* < 0.05) (Figure 1). However, no strain differed significantly from the control in post hoc comparisons using the Dunn test (Table S8). Exploratory pairwise comparisons between groups identified a significant difference between SAM4 without silver stress and SAM5 with silver stress, suggesting that the overall data reflected variation among treatment groups rather than a shift relative to the control (Figure 1). However, SAM5 with silver had the lowest mean depth in the dataset and SAM4 without silver was among the highest (CNK fractions 0.083 vs. 0.087), so this variation was not considered a treatment difference. Similarly, such secondary analysis indicated significant treatment group variation for SAM2 without silver stress and SAM1 with silver stress for global 6mA methylated fractions, which is also indicative of treatment group differences (Figure 1). Additionally, CNK methylated fractions vary with sequencing depth, so small differences in global 4mC between conditions were not interpreted as treatment effects (for example, treated about 0.084 vs. untreated about 0.086).

In the absence of silver stress, one replicate of SAM3 deviated from the dominant 6mA global methylation pattern observed in other strains. This replicate was included in all downstream analyses for the enrichment tests and the comparisons of global methylation fractions. The deviation of the 6mA global methylation of this replicate was determined to be likely due to the presence of a less abundant, minor motif, namely GACYNVNBND, with a very low methylation fraction at ~0.06 (Table S2). To confirm whether the outlier reflected a broad methylome shift or a motif-specific effect, we conducted a sensitivity analysis where we recalculated the global methylation after excluding the low-fraction motif (GACYNVNBND). The replicate no longer appeared as an outlier, indicating that the deviation was driven primarily by that motif rather than by genome-wide methylation loss. The results obtained from this sensitivity analysis also did not change the overall Mann–Whitney U and Kruskal–Wallis results of all 11 strains and their replicates and treatments compared to the control. Hence, this outlier likely reflects a motif-composition effect in the global methylation, where a low-fraction motif disproportionately influenced the replicate-level average. This therefore may represent a threshold-sensitive methylation call where a low-fraction motif was detected at a lower level in one replicate and therefore reduced the calculated global fraction. However, this did not lead to loss of the dominant methylation motifs, which remained highly methylated and may simply reflect biological heterogeneity in methyltransferase activity affecting a minor or partially methylated motif, rather than a global loss of methylation. Also, because the motif showed low methylation fraction and greater replicate variability than the dominant motifs, it was interpreted as a lower-confidence or condition-sensitive motif rather than a stable core methylation signal.

**Figure 2:**
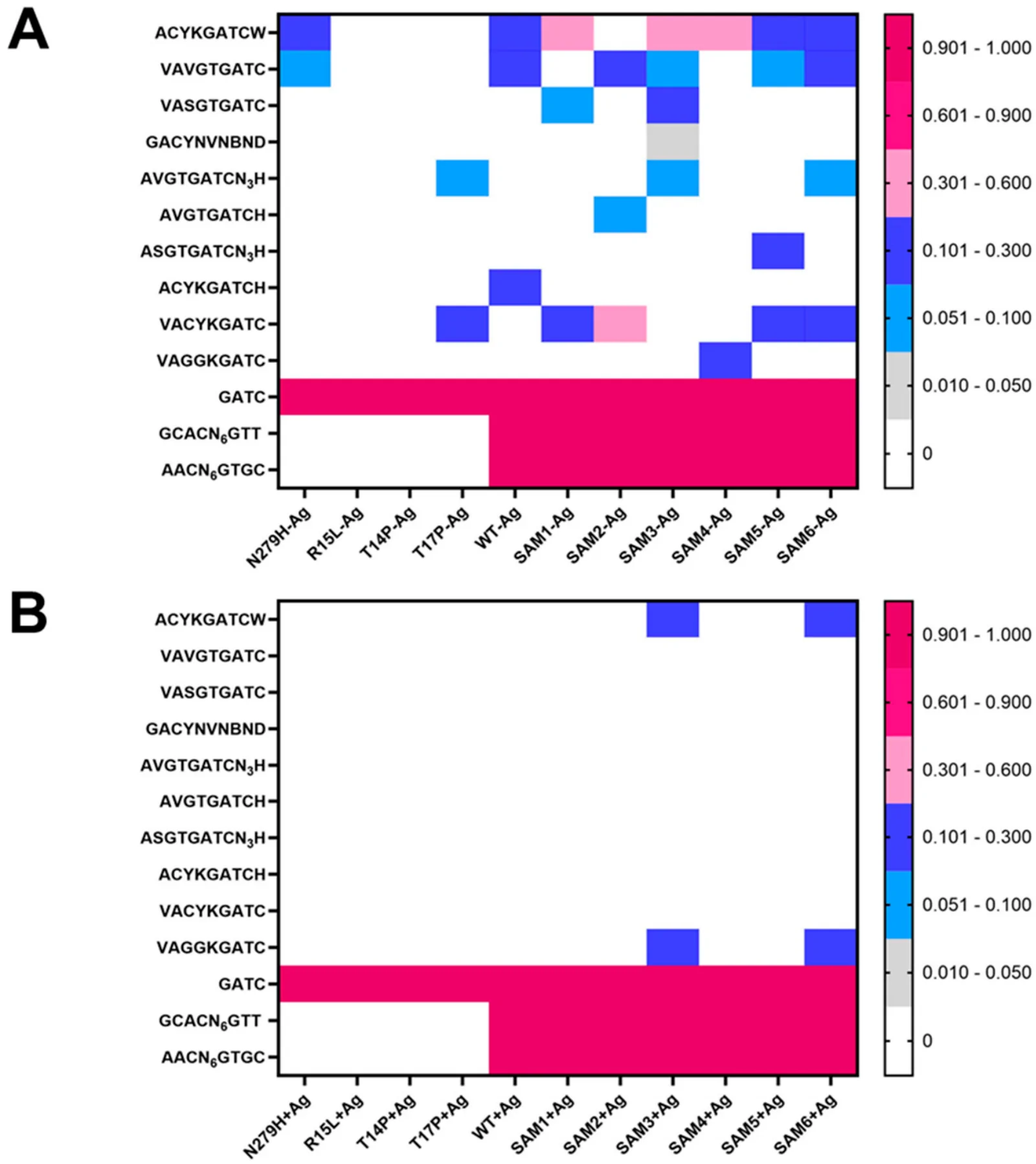
Heat map of motif-specific 6mA methylation fractions across strains and treatment conditions. Heat maps show the mean methylation fraction for each motif in each strain, averaged across biological replicates for (**A**) without silver treatment and (**B**) with silver treatment. Color bins are non-linear and were selected to improve visual discrimination across the wide range of methylation fractions, particularly among near-saturated motifs. Motifs are ordered by approximate global prevalence across strains and conditions rather than alphabetically or by sequence similarity, to facilitate comparison across the numerous degenerate motifs.

Among 6mA methylation motifs, the AACN_6_GTGC/GCACN_6_GTT and GATC motifs consistently showed complete or near-complete methylation across multiple strains (Figure 2). The GATC motif was detected in all strains and replicates regardless of treatment, while the bipartite AACN_6_GTGC/GCACN_6_GTT motif was present in all replicates of the wild-type and silver-adapted populations both with and without silver exposure. The bipartite AACN_6_GTGC/GCACN_6_GTT motif was also absent across all *cusS* mutants, which can be attributed to a shared single-base-pair deletion in *hsdS*, observed across all *cusS* recombineered clones. This phenomenon is examined in more detail in Section 4 of this paper. In contrast, extended degenerate motifs containing a conserved GATC core or a conserved GAC prefix were also detected in the wild-type, two *cusS* mutants and all six adapted populations (Figure 2).

However, after silver exposure, most of these degenerate motif signals were not detected in the wild-type and most silver-adapted populations. Specifically, none of the minor motifs were detected except the ACYKGATCW and VAGGKGATC motifs in both SAM3 and SAM6. The lack of detection of several degenerate, low-abundance motif calls after treatment across multiple strains and replicates is reported as an observation since silver-treated samples were sequenced to a lower mean depth (Table S1) and detection of low-fraction motifs depends on coverage and calling thresholds (Figure 2). Because low-abundance degenerate motifs may be more sensitive to coverage, kinetic signal strength, and motif-calling thresholds, these changes were considered putative.

**Figure 3:**
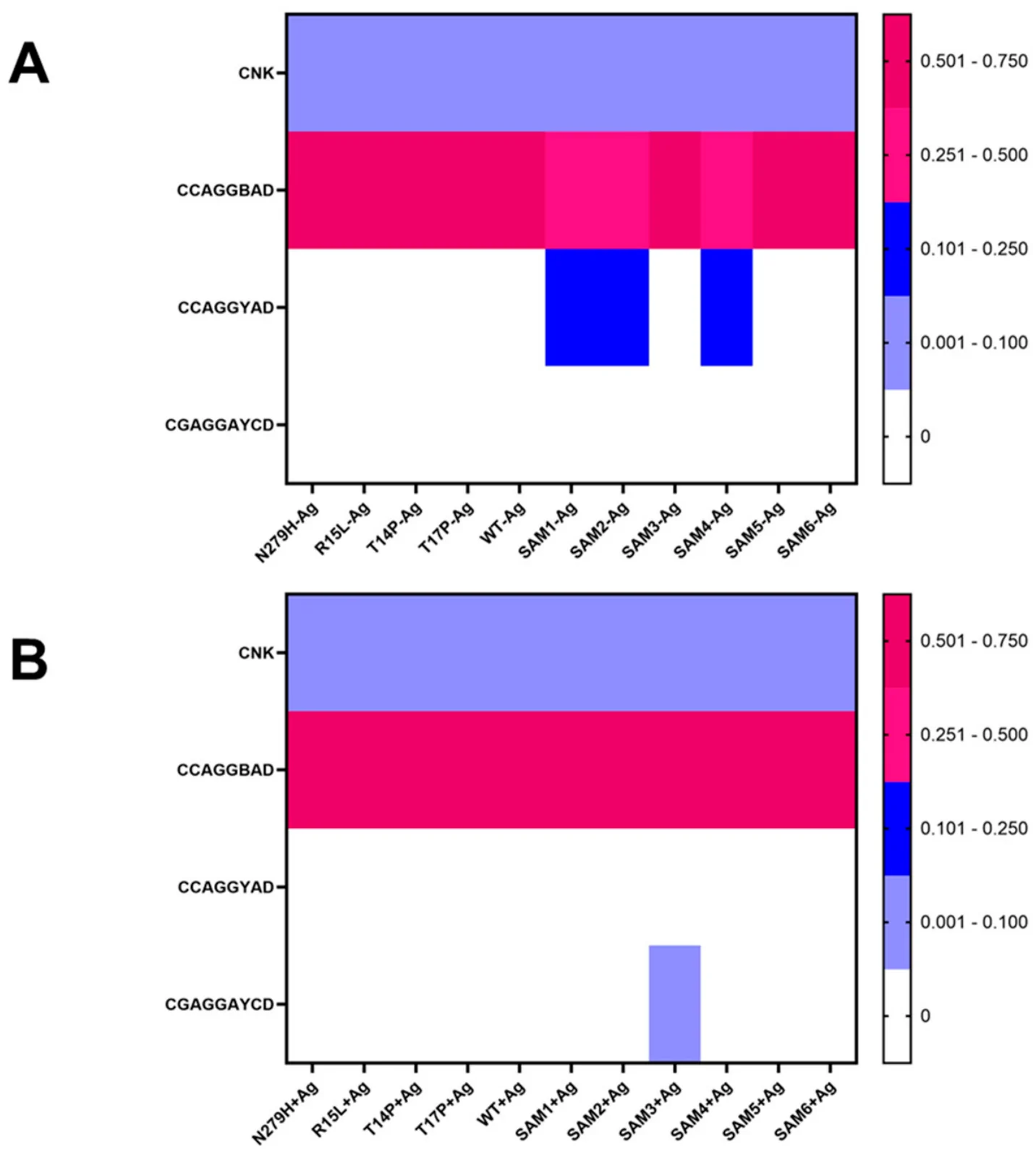
Heat map of motif-specific putative 4mC methylation fractions across strains and treatment conditions. Heat maps show the mean methylation fraction for each motif in each strain, averaged across biological replicates for (**A**) without silver treatment and (**B**) with silver treatment. Color bins are non-linear and were selected to improve visual discrimination across the wide range of methylation fractions, particularly among near-saturated motifs. Motifs are ordered by approximate global prevalence across strains and conditions rather than alphabetically or by sequence similarity to facilitate comparison across the numerous degenerate motifs.

The putative 4mC methylation fractions detected among our strains were CNK, CCAGGBAD, CCAGGYAD and CGAGGAYCD. Without silver exposure, the CNK motif was present in all strains and replicates, but its low methylation fraction (~0.08–0.09) suggests a conserved partial methylation state rather than strong population-wide methylation (Figure 3). The CCAGGBAD was also detected in all strains; however, it was not universally detected across all replicates. Specifically, this motif was not detected in one replicate each of SAM1, SAM2 and SAM4, indicating variable detection within these strains. Interestingly, CCAGGYAD was detected only in the same replicates of SAM1, SAM2 and SAM4 in which CCAGGBAD was not observed. After silver exposure, the presence of the CNK motif remained consistent across all strains and replicates without any changes to the pattern of low methylation fractions (~0.08–0.09) observed before treatment. The CCAGGYAD motif was not detected in all strains and replicates after silver treatment, whereas all strains and replicates consistently harbored the CCAGGBAD motif. The only other motif present in silver-treated *E. coli* was CGAGGAYCD in one replicate of SAM3. This minor motif was included in all chi-square, Mann–Whitney U tests and Kruskal–Wallis tests, and did not lead to any deviation from general 4mC methylation trends. We also conducted a sensitivity analysis where we recalculated the global methylation after excluding the low-fraction motif (CGAGGAYCD). The results obtained did not change the overall Mann–Whitney U results of the strain whose replicate contained the motif, nor did it change the Kruskal–Wallis results of all 11 strains and their replicates and treatments compared to the control. Since this minor motif appeared only once in the dataset and in one post-treatment replicate, we reported it transparently but do not consider it as primary evidence for treatment-associated methylome remodeling. Its occurrence may reflect replicate-specific biology or threshold-sensitive motif detection.

From our analyses, our overall results support motif-level methylome redistribution with relative stability of global methylation fractions. These results are not contradictory, as each test answers a different biological question. The chi-square test is sensitive to changes in the distribution of methylated motif counts, while Mann–Whitney and Kruskal–Wallis tests assess whether replicate-level methylation fractions differ between or among groups. Therefore, significant chi-square results indicate that the composition of methylated motifs changed, but the non-significant Mann–Whitney results indicate that these changes did not produce strong pairwise shifts in overall methylation fractions. The significant Kruskal–Wallis results suggest broader group-level differences across all samples, even though individual pairwise comparisons were not strong enough to be significant. Heat maps, on the other hand, showed that before treatment, methylated motif profiles were more heterogeneous, with variable detection of several minor/low-fraction motifs across samples. Following treatment, the heat map showed a more homogeneous motif profile, indicating fewer low-abundance motifs were detected after treatment, while the dominant methylation systems were unchanged.

## 4. Discussion

This study provides a genome-wide characterization of DNA methylation in *Escherichia coli* K-12 MG1655. Our interpretation of treatment-associated methylation changes was guided by motif-level quality metrics, with a detected genome size of approximately 4.64 Mb, >400× mean coverage and % bp ≥ Q30 of at least 97%. High-fraction motifs with strong coverage and elevated IPD ratios were considered high-confidence methylation signals, whereas low-abundance, degenerate or inconsistently detected motifs across replicates were interpreted cautiously. Our analyses revealed that 6mA, putative 4mC and several modified bases were present within our dataset, consistent with data detectable using PacBio Single-Molecule Sequencing in Real Time and bacterial DNA methylation classes reported in the scientific literature. The most prevalent methylation form observed in bacteria is 6mA [24,25,44], while 4mC is also present [24,25,44]. Most of the eleven strains exhibited near-complete global 6mA methylation and minimal variance, which is suggestive of strong enzymatic regulation under our conditions. Global mean 6mA methylation fractions ranged from ~0.69 to nearly 1.00, driven primarily by two main motifs. The main 6mA motifs present in our dataset were the GATC and the bipartite AACN_6_GTGC/GCACN_6_GTT motifs. The GATC motif was almost fully methylated (methylated fractions > 0.99 but <1) and conserved across all our strains/conditions, which reflects high activity of the Dam methyltransferase. This methylation type is both typical and strong in *E. coli*. Recent literature supports this phenomenon with genome-wide Dam GATC methylation and near-complete methylation occurring over extended periods of time during *E. coli* K-12 growth [27], its prevalence in *E. coli* and its responsibility for methylating 99% of GATC motifs [45].

Interestingly, the prevalence of the GATC motif and its near-complete methylation were consistent across all strains, both in the presence and absence of low levels of silver stress. In previous research, it has been reported that the GATC motif remained stable despite changing conditions or stress. For example, Westphal et al. showed that in an *E. coli* study, practically all GATC sites were significantly methylated over time, with near-complete methylation persisting throughout the early stationary phase through long-term stationary phase and death-phase conditions [27]. The researchers viewed these findings as proof that methylation is prioritized despite nutrient scarcity [27]. Bourgeois et al. also describe a highly stable methylome system, particularly highly resistant GATC motifs, that is decoupled from the transcriptome in *Salmonella enterica* serovar Typhimurium under different media conditions [46]. This was despite broad changes occurring in the transcriptome under the same conditions [46]. Under more adverse conditions in another study, *Salmonella enterica* serovar Typhimurium was exposed to hydrogen peroxide, where oxidative stress did not alter total 6mA GATC levels [31]. Cohen et al. also conclude that the bacterial GATC methylome plays a role in antibiotic stress survival despite remarkable stability of the GATC motif to ampicillin exposure [47]. Indeed, the GATC motif is a highly, efficiently methylated part of the bacterial DNA methylome and its critical roles in DNA repair [24,48], DNA replication [49,50] and some reported stress environments/conditions [27,47] likely contribute to its persistence and resilience in *E. coli* genomes.

In our study, we also detected AACN_6_GTGC/GCACN_6_GTT, a bipartite, asymmetric degenerate motif pair, which was conserved across our wild-type *E. coli* and all our SAMs. There was consistent saturated or near-saturated methylation of these motifs (methylated fractions 0.99–1), underscoring their likely biological significance. In recent work, McIntyre et al. identified this motif pair among the known methylated motifs in *E. coli* with estimated modification fractions above 90%, supporting that these sites are typically highly methylated in *E. coli* K-12-related backgrounds [51]. Mehershahi et al. likewise described EcoKI as methylating this motif in *E. coli* and noted that EcoKI methylates many hundreds of distinct genomic sites, indicating that this recognition pattern is not rare [52]. In some *E. coli* strains, especially *E. coli* K-12 MG1655 and related lab strains, AACN_6_GTGC/GCACN_6_GTT is a major EcoKI-associated Type I restriction-modification motif [53] that is important for genome defense [52]. Taken together, these studies support the interpretation that this bipartite motif pair represents a canonical and typically strongly methylated feature of *E. coli* methylation architecture. This research adds to this body of knowledge by showing that this motif is retained in independent lineages of the parental wild-type of *E. coli* under and without silver stress and also shows how that trend differs from methylation patterns observed in engineered mutants of the same strain and under the same conditions.

While most of our strains had near-complete methylation, one replicate of SAM3 in the absence of silver stress appeared to fall outside the dominant 6mA global methylation pattern, owing largely to differences in a less abundant, minor motif, namely GACYNVNBND. Because the dominant motifs of all strains, including all three replicates of SAM3, remained broadly consistent, this deviation is more likely to reflect variation in lower-abundance motifs or replicate-level noise than a major shift in the overall methylome. We therefore interpret this cautiously as replicate-level variability of SAM3 rather than a stable strain-wide methylation shift. Also, the lower global methylation fraction observed in one replicate appeared to be driven primarily by a low-fraction motif rather than by a broad reduction across dominant methylation systems. Major motifs remained highly methylated and consistent across replicates, whereas the low-fraction motif showed greater replicate-level variability. This suggests that the outlier reflects sensitivity of the global methylation to motif-specific variation, possibly due to partial methylation, threshold-dependent motif detection or biological heterogeneity in methyltransferase activity. Therefore, the replicate was retained in our analyses, but the affected motif was interpreted cautiously as a variable or putative methylation signal rather than as evidence of global methylome disruption.

Five samples of SAM4 assembled to ~4,624,965–4,624,967 bp: namely, two replicates of SAM4 without silver and all three replicates of SAM4 with silver. However, one replicate of SAM4 without silver assembled to about 4,640,063 bp. Comparative analysis showed that the approximately 15 kb sequence absent from the shortened SAM4 assemblies corresponded to the e14-associated prophage locus, which has been found in some *E. coli* strains [54]. This inference was supported by pairwise NCBI BLASTn (https://blast.ncbi.nlm.nih.gov/Blast.cgi?PROGRAM=blastn&PAGE_TYPE=BlastSearch&LINK_LOC=blasthome; accessed on 27 September 2026) against the MG1655 e14 reference region, PHASTEST prophage prediction (https://phastest.ca/; accessed on 27 September 2026) and Bakta/JBrowse annotation (https://jbrowse.org/jb2/; accessed on 28 September 2026) of characteristic e14-associated genes, including *mcrA*, *pinE*, *tfaE*, *croE*, *cohE*, *xisE*, *intE*, *lit* and multiple *ymf* genes [55]. These analyses independently showed that the locus was retained in the wild-type and the normal-length SAM4 assembly but absent from the shortened SAM4 assemblies. The loss of the e14-associated region across five SAM4 assemblies, its retention in the normal-length SAM4 replicate and the corresponding contraction of the flanking *icd* neighborhood strongly support a genuine deletion/excision of this region in the affected samples. Also, because all SAM4 cultures originated from the same glycerol stock, the occurrence of e14 excision in five cultures and retention in one is consistent with heterogeneity in the original stock. Stress conditions also have been known to lead to excision of the e14-associated region in bacteria, as reported with antibiotic exposure [56,57], oxidative stress [57] and ionizing radiation [58]. Hence, it is possible that silver adaptation could have led to or contributed to the excision of the e14 region in some populations of the SAM4 lineage. However, this study cannot conclusively establish this. Methylation fractions were calculated from the motif sites present in each sample’s assembly. Due to the fact that the shortened SAM4 genomes lack the e14 region, their methylation fractions describe a slightly different set of genomic sites than those of the other samples. For example, the shortened assemblies contain 84 fewer GATC sites (0.22% of 38,246), a difference that is unlikely to materially affect comparisons of SAM4 methylation fractions with those of other strains.

The most consistent putative 4mC motifs present across our strains were the CNK and CCAGGBAD motifs, followed by CCAGGYAD and CGAGGAYCD motifs. Upon searching the scientific literature, we could not find any published reports for these methylated motifs. We also searched within REBASE (the restriction enzyme database) [59] and found no records for CNK, CCAGGBAD and CGAGGAYCD. We did find a couple of records of the CCAGGYAD motif in REBASE, with notes indicating that the motif is likely a miscall for CCWGG [59]. We suspect that other researchers have also encountered the occurrence of ambiguous methylation motifs in their sequencing data. And if so, we believe that increased reporting of this phenomenon in published papers will help clarify its prevalence, whether rare or common within the context of DNA methylation. This practice ultimately will benefit researchers across the field. Because the degenerate cytosine-containing CCAGGBAD and CCAGGYAD motifs contain a CCAGG core, these degenerate motifs are not likely to be definite 4mC motifs but possibly putative 4mC motifs. The CCWGG motif is widely recognized as connected to Dcm methyltransferases and 5mC motifs [60,61]. Our CCAGGBAD and CCAGGYAD motifs could also be considered to have a Dcm-associated sequence with an additional degenerate sequence. Since metrics of sequencing quality of these motifs are high, e.g., IPD ratio of >1, with moderate consistent methylation fractions (~0.6–0.7), and the IPD ratios of the dominant GATC and AACN_6_GTGC/GCACN_6_GTT motifs ranged from 4 to approximately 6, the kinetic signals detected during our SMRT sequencing set are real. Because native 5mC was not a supported modification class in the ipdSummary analysis used in this study, a kinetic signal arising from an unsupported modification could potentially be assigned to one of the supported modification classes. Thus, the classification of CCAGGBAD and CCAGGYAD as 4mC should be regarded as putative rather than as definitive evidence of their chemical identity. Notably, both motifs contain an exact CCAGG core, corresponding to the W = A variant of the canonical Dcm recognition sequence CCWGG, with no mismatches within the five-base Dcm core. Each motif additionally contains three downstream positions beyond the canonical recognition sequence, comprising BAD in CCAGGBAD and YAD in CCAGGYAD, respectively. Furthermore, since 5mC is not a motif known to be strongly detected by SMRT sequencing, but is possible using higher sequencing coverage (>250×), as is the case with our dataset, the results from our dataset suggest that some kinetic signatures classified as putative 4mC may correspond to alternative cytosine modifications that can be validated by secondary orthogonal methods. It should also be noted that other CCAGG-extended motifs have been identified in some previous research as 4mC [31,62].

The putative 4mC CNK motif displayed consistently low methylation fractions across all strains, both without silver stress and under silver stress conditions. This motif is also not reported in the scientific literature to the best of our knowledge; hence, we describe this motif cautiously as a putative 4mC degenerate cytosine-context motif. Although these low-fraction signals were far less prominent than the dominant methylation features, their reproducible detection suggests that they do not merely reflect stochastic noise or isolated sample-specific events. Rather, they may represent stable partial methylation states maintained across our dataset. Nevertheless, no direct functional inference can be made from stability alone.

In our study, differences in motif detection were also observed across conditions, with selected motifs detected preferentially in control or stress-exposed samples. For example, in putative 4mC motifs, the moderately methylated CCAGGBAD motif was conserved across most strains prior to exposure to silver, with the exception of one replicate each of SAM1, SAM2 and SAM4. In replicates where this motif was not detected, the CCAGGYAD motif was present. After silver exposure, however, the CCAGGYAD motif in these replicates was not detected in the same replicates; rather, the CCAGGBAD motif was then detected across those replicates such that this methylation signal was consistent across all our strains. The inconsistent pre-treatment detection of this moderate motif, followed by uniform post-treatment detection across replicates, suggests treatment-associated convergence of a motif-specific methylation signal. It is possible that the responsible methyltransferase system was variably active before treatment but became stabilized or more consistently active after exposure. An alternative explanation is that the motif was present at a threshold-sensitive level before treatment and became more consistently detectable after treatment. Thus, this pattern could be interpreted as a putative treatment-associated stabilization or detection of a moderate methylation motif rather than definitive evidence of a newly acquired global methylation system.

Similarly, in our 6mA motifs, detectable methylation signals changed depending on the treatment conditions of the bacteria. The GATC motif was conserved across all strains, and the AACN_6_GTGC/GCACN_6_GTT motif was conserved across the wild-type and all SAMs, all with and without silver exposure. It should also be noted that across most strains, less dominant degenerate motifs containing either a GATC core or a GAC core were present. Extended degenerate motifs with either a GATC or GAC core have been reported in multiple published works. Murray et al. describe the incomplete methylation of the RGATCY motif in *Chromohalobacter salexigens*, which is distinct from the GATC motif in the same bacterium [63]. The same extended motif was also detected via SMRT sequencing in *Chromohalobacter salexigens* and *Helicobacter pylori* J99 in the work of Beaulaurier et al. [64]. Likewise, in more recent research using SMRT sequencing, highly methylated RGATCY motifs were also reported in *Acinetobacter baumannii* [65] and in *Burkholderia cepacia* [66]. The extended GATC(N)_6_GTC motif was described as related to a type 1 restriction-modification system in *Pseudomonas aeruginosa* PAO1 [67]. GAC, GAC-like, GATC and GATC-like motifs have also been observed in *Klebsiella pneumoniae* as CGACCN_4_TGG and CCAN_4_GATCA [68], CANCATC and GAGACC in *Escherichia coli* ST131 [69] and the bipartite GACNNNNNTGA/TCANNNNNGTC motif in *Bacteroides fragilis* [70]. Before silver treatment, most strains exhibited a broader range of detected degenerate motifs. The silver-treated strains exhibited a comparatively more homogeneous motif-detection profile, characterized by fewer detected low-abundance degenerate motifs. The reduced diversity of detected low-abundance degenerate motifs following silver exposure is consistent with the hypothesis that stress may reduce methylome complexity. However, because silver-treated samples also had systematically lower sequencing depth, coverage-dependent motif detection and caller threshold effects cannot be excluded as alternative explanations for this observation. The lack of detection of low-abundance degenerate motif calls after treatment may reflect variation in methylation signal strength rather than loss of the DNA motif itself. These motifs were therefore interpreted as putative, threshold-sensitive methylation signals rather than definitive treatment-regulated motifs. Studies explicitly tracking bacterial populations in the presence and absence of stressors with reports of condition-specific methylation dynamics are rare. To mention just a few studies, Zimmerman et al. demonstrate the plasticity of the *Helicobacter pylori* methylome and its acid-responsiveness [33]. They reported sequences that are methylated at a pH of 5 but not methylated at a pH of 7. In another study, Thakur et al. describe environmental stress, which was associated with both loss of some detectable motif signals and appearance of other motif signals [71].

Another interesting observation across our dataset was that GATC motifs are conserved across all strains, which indicates their biological significance. This is even more important seeing that this motif was not altered even in engineered mutants whose genetic sequence was altered. It should be noted that the four *cusS* mutants differ from one another in the single-nucleotide substitution each carries in *cusS.* This suggests that this motif is possibly very important in *E. coli* and that, based on our conditions, it will likely remain conserved in single-nucleotide engineered mutants or similar mutants where extensive DNA sequences remain unaltered. The exception to this in engineered strains would be if the GATC motif is directly targeted during engineering. The stability of GATC before and after treatment can also be attributed to its abundance in the genome and the importance of the Dam methylation system in replication timing, mismatch repair, and genome maintenance [19,47,72]. However, the bipartite AACN_6_GTGC/GCACN_6_GTT motifs were consistently present in both wild-type and our six SAMs. This also suggests that mutations, while *E. coli* adapts, favor retaining not just the GATC motif, but also favor retaining the EcoKI-associated Type I restriction-modification motif inherited from its parental strain. However, this does not imply that the absence of this motif will affect cell viability. It should also be noted that these six SAMs are unique from each other, although having different levels of similarity and difference. In the study from which the single *cusS* mutants were generated via pORTMAGE recombineering, all recombineered clones harbored the same single-base pair deletion within *hsdS* [32]. This was an unintended off-target deletion in *hsdS* as a result of pORTMAGE engineering. Although the same off-target deletion was present in all of our *cusS* mutants, the mutants were not genetically identical because each carried a different intended single-nucleotide substitution in *cusS*. This *hsdS* mutation was not observed in the studies where the silver-adapted mutants were generated and in other studies using the same parental *E. coli* K-12 MG1655 strain [7,8,12,73,74]. The bipartite AACN_6_GTGC/GCACN_6_GTT motifs are a classic signature of EcoKI-like type I R-M systems, which require a multi-subunit complex of HsdM, HsdS and HsdR [35,53,75,76,77]. Any compromise within *hsdS,* as observed for the *cusS* mutants, could lead to an EcoKI system that is inactive or weak and below the detection threshold. For more context, HsdS is required to bring HsdM together into a functional methyltransferase and further to recruit HsdR for restriction since *hsdS* encodes the specific DNA-recognition subunit [76] and is responsible for sequence specificity [53]. Previous studies have shown that deletion of a single nucleotide in *hsdS* altered the DNA sequence specificity of a type I Restriction-Modification System [78]. Mutating or recombining the *hsdS* gene is enough to change how the entire Type I system recognizes and modifies DNA [52], and duplicated/distinct *hsdS* specificity genes can switch Type I Restriction-Modification specificity and cause genome-wide methylation differences [79]. Thus, differences in *hsdS* integrity or activity are a plausible mechanistic explanation for the absence of the EcoKI-associated bipartite methylation motif in the *cusS* mutants compared to the SAMs. This *hsdS* mechanistic explanation is only applicable to the complete absence of AACN_6_GTGC/GCACN_6_GTT motif signals in our *cusS* mutants, with and without silver treatment, and should not be confused with other GATC/GAC-core degenerate motifs in our dataset. The GATC/GAC-core degenerate motif signals were detected in only one untreated replicate each of N279H (replicate 3) and T17P (replicate 1), in no treated replicate, and in no R15L or T14P replicate. That sporadic detection fits threshold-sensitive calling of low-fraction motifs and contrasts with the complete absence of the AACN_6_GTGC/GCACN_6_GTT motif in every *cusS* mutant replicate.

Our analysis indicates that the global DNA methylome of *E. coli* K-12 appears largely stable across the strains and silver stress conditions tested, although site-specific methylation dynamics at individual regulatory loci were not assessed in this study. For poorly methylated motifs such as CNK, their biological relevance remains uncertain and will require targeted follow-up studies in future work. Similarly, CCWGG-extended degenerate motifs will be investigated further in future work. Because the modified bases do not provide confident chemical resolution, we treated these signals as unresolved modification calls and did not use them for biological inference. Hence, in our future work, we will use orthogonal approaches to determine the chemical identity and biological significance of these unresolved signals across all strains. These approaches will also be applied to putative 4mC identified across our strains. Separately, the structural differences identified in SAM4 and SAM6 may warrant further investigation to determine their genomic and potential functional consequences and potential relationship to strain-specific methylation patterns.

## 5. Conclusions

This study expands current understanding of the bacterial DNA methylome by characterizing 6mA and putative 4mC methylation across eleven *Escherichia coli* K-12 MG1655 strain backgrounds. To our knowledge, this is the first study to compare genome-wide DNA methylation among wild-type *Escherichia coli* K-12 MG1655, *cusS* mutants and silver-adapted populations, with and without silver exposure. The identification of dominant and reproducibly detected methylated motifs establishes a baseline methylome resource for comparing genetic variants, adapted populations and stress-exposed conditions. Under our conditions, we also identified non-conserved and strain-associated motifs, with additional variation in motif detection across silver-treatment conditions. Despite genetic differences among strains and exposure to low-level stress, major methylation motifs remained largely conserved across biological replicates, suggesting that core methylation systems in *E. coli* are highly stable and resilient. Our analyses characterize genome-wide methylation patterns at the motif level, while site-specific dynamics at individual regulatory loci were not assessed here and will be investigated in future work. Furthermore, in this study, variation among lower-abundance or putative motifs highlights the potential for more subtle motif-specific differences that may reflect biological and/or technical sources of variation. Comparing our findings with those also reported in the literature, particularly the resistance of the DNA methylome to change across multiple strains and silver stress conditions, we speculate that under low-level silver stress in *E. coli* K-12, the dominant DNA methylation features appear remarkably resilient, consistent with a structural rather than condition-responsive role for the core methylome. These findings provide a foundation for future work on the biological significance of bacterial DNA methylation in the presence and absence of environmental stressors and the implications of such methylation patterns for bacterial acclimation and adaptation.

## Figures and Tables

**Table 1:** Chi-square analysis of 6mA global-count distributions across strains and treatment conditions.

Strain	With Silver?	Chi-Square	df	*p*-Value	Effect Size (Cramér’s V)	Significant?
N279H	No	3750	5	<0.0001	0.1266	Yes
R15L	No	4146	5	<0.0001	0.1332	Yes
T14P	No	4147	5	<0.0001	0.1332	Yes
T17P	No	4427	7	<0.0001	0.1376	Yes
SAM1	No	631.4	7	<0.0001	0.05153	Yes
SAM2	No	1081	7	<0.0001	0.06738	Yes
SAM3	No	1635	8	<0.0001	0.08267	Yes
SAM4	No	491.6	6	<0.0001	0.04549	Yes
SAM5	No	531.9	7	<0.0001	0.04728	Yes
SAM6	No	680.2	7	<0.0001	0.05346	Yes
Wild-type	Yes	650.2	5	<0.0001	0.05235	Yes
N279H	Yes	4146	5	<0.0001	0.1332	Yes
R15L	Yes	4146	5	<0.0001	0.1332	Yes
T14P	Yes	4147	5	<0.0001	0.1332	Yes
T17P	Yes	4147	5	<0.0001	0.1332	Yes
SAM1	Yes	650.2	5	<0.0001	0.05235	Yes
SAM2	Yes	650.2	5	<0.0001	0.05235	Yes
SAM3	Yes	494.1	6	<0.0001	0.04562	Yes
SAM4	Yes	648.8	5	<0.0001	0.05232	Yes
SAM5	Yes	650.2	5	<0.0001	0.05235	Yes
SAM6	Yes	493.6	6	<0.0001	0.04559	Yes

Chi-square tests were used to assess whether the distribution of 6mA global motif counts differed between control (wild-type without silver treatment) and strains subjected to silver stress at *p* < 0.05. Also, note that degrees of freedom ranged from 5 to 8 among strains and conditions because the number of distinct methylated motifs detected differed across strains and conditions. These tests involve different motif categories; hence, their chi-square statistics and effect-size magnitudes should be compared cautiously across rows.

**Table 2:** Chi-square analysis of putative 4mC global-count distributions across strains and treatment conditions.

Strain	With Silver?	Chi-Square	df	p-Value	Effect Size (Cramér’s V)	Effect Size (Phi Coefficient (φ))	Significant?
N279H	No	0.02693	1	0.8696	NA	0.0002	No
R15L	No	0.003427	1	0.9533	NA	0.00007131	No
T14P	No	1.157	1	0.2822	NA	−0.001306	No
T17P	No	0.5331	1	0.4653	NA	−0.0008886	No
SAM1	No	1193	2	<0.0001	0.04218	NA	Yes
SAM2	No	1163	2	<0.0001	0.04159	NA	Yes
SAM3	No	0.0006827	1	0.9792	NA	0.0000319	No
SAM4	No	1185	2	<0.0001	0.04186	NA	Yes
SAM5	No	1.475	1	0.2246	NA	−0.001483	No
SAM6	No	1.201	1	0.2732	NA	−0.001333	No
Wild-type	Yes	4.827	1	0.028	NA	0.002696	Yes
N279H	Yes	3.534	1	0.0601	NA	0.002308	No
R15L	Yes	4.349	1	0.0370	NA	0.002557	Yes
T14P	Yes	3.579	1	0.0585	NA	0.002314	No
T17P	Yes	3.080	1	0.0793	NA	0.002146	No
SAM1	Yes	3.937	1	0.0472	NA	0.002434	Yes
SAM2	Yes	2.128	1	0.1446	NA	0.001787	No
SAM3	Yes	65.80	2	<0.0001	0.009926	NA	Yes
SAM4	Yes	3.397	1	0.0653	NA	0.002259	No
SAM5	Yes	4.035	1	0.0446	NA	0.002471	Yes
SAM6	Yes	2.731	1	0.0984	NA	0.002025	No

Chi-square tests were used to assess whether the distribution of 4mC global motif counts differed between control (wild-type without silver treatment) and strains subjected to silver stress at *p* < 0.05. The negative sign of the phi coefficient of some samples means the association of the treatment goes in the opposite direction of the control. In such cases, the absolute value of phi was used to interpret effect-size magnitude, while the sign indicates direction of the effect. Also, note that degrees of freedom ranged from 1 to 2 among strains and conditions because the number of distinct methylated motifs detected differed across strains and conditions. These tests involve different motif categories; hence, their chi-square statistics and effect-size magnitudes should be compared cautiously across rows. Note: NA (not applicable).

## Data Availability

Data for this project have been deposited and the public access code for the Bioproject is PRJNA1503007 with SRA accession numbers SRR40336523–SRR40336588.

## Supplementary Materials

The following supporting information can be downloaded at https://www.mdpi.com/article/10.3390/microorganisms14102369/s1, Table S1: sequencing quality metrics; Table S2: per-sample methylated motif calls and supporting SMRT kinetic quality metrics; Table S3: chi-square analysis of 6mA global-count distributions across strains and treatment conditions at the replicate level; Table S4: chi-square analysis of 4mC global-count distributions across strains and treatment conditions at the replicate level; Table S5: Mann–Whitney U tests of 6mA global methylation fractions across strains and treatment conditions; Table S6: Kruskal–Wallis tests of 6mA global methylation fractions across strains and treatment conditions; Table S7: Mann–Whitney U tests of 4mC global methylation fractions across strains and treatment conditions; Table S8: Kruskal–Wallis tests of 4mC global methylation fractions across strains and treatment conditions.
